# Vascular Oxidative Stress: Pharmacological and Nonpharmacological Approaches

**DOI:** 10.1155/2018/8539350

**Published:** 2018-07-22

**Authors:** Valeria Conti, Maurizio Forte, Albino Carrizzo

**Affiliations:** ^1^Department of Medicine, Surgery and Dentistry “Scuola Medica Salernitana”, University of Salerno, Baronissi, 84081 Salerno, Italy; ^2^Department of AngioCardioNeurology, IRCCS Neuromed, Pozzilli, 86077 Isernia, Italy

Cerebral and cardiovascular diseases represent a big public health problem and a leading cause of mortality in the world. In more than 40% of cases, they occur before 75 years of age. The most common forms of CVDs are ischemic heart disease, stroke, and arterial hypertension.

The cardiovascular risk is continuous during the life, and all people have to reckon cardiovascular risk, which depends on the combination of different factors (age, sex, smoking and diet habits, high blood pressure, and cholesterolemia).

The modifiable risk factors should be tackled with interventions promoting correct lifestyles and appropriate pharmacological therapy. Right lifestyles must be encouraged in the population starting from young age in order to maintain a favourable cardiovascular risk profile as long as possible.

Oxidative stress results from an imbalance between oxidizing compounds and antioxidant resources provided by the endogenous defense system. The generation of oxidizing compounds occurs physiologically in response to insults and tissue repair processes. On the other hand, an improper activation of oxidative processes can be chronically present in pathological conditions, such as arterial hypertension and diabetes mellitus, contributing to the oxidative damage. Vasorelaxant molecules, such as nitric oxide (NO), hydrogen sulfide (H_2_S), and prostaglandin I2 (PGI_2_), play a fundamental role in the regulation of vascular tone and are especially damaged by excessive oxidative stress production.

The present special issue includes papers that explore the effects of the oxidative stress on the onset and progression of the cerebral and cardiovascular diseases and the mechanisms helpful to slow down their occurrence.

It has been shown that radical oxygen species, including hydrogen peroxide hydroxyl radical, peroxynitrite, and superoxide anion, act as molecule signal that modulate multiple cellular responses. The main sources of O2^•−^ generated in the organism are NOx. These oxidases produce O2^•−^ from oxygen using NADPH as an electron donor. The NOX family is the only family of enzymes known with the function of producing ROS that can be activated by different stimuli. NADPH oxidase-derived ROS act as intracellular second messengers by activating several redox signalling cascades implicated in VEGFR2 autophosphorylation, EC migration, angiogenesis, and proliferation, but molecular mechanisms responsible for NADPH oxidase activation and the function of ROS in redox signalling linked to angiogenesis remain unclear.

F. Cattaneo and colleagues in an experimental model of human endothelial cell line reported that the activation of formyl-peptide receptors (FPRs) induces superoxide generation as a consequence of MEK- and PKC-dependent phosphorylation of the regulatory subunit p47^phox^, demonstrating that ROS generation is regulated by the binding of N-formyl-methionyl-leucyl-phenylalanine (N-fMLP) to FPR1 and that produced ROS mediate Flk-1/KDR transactivation, playing a crucial role in VEGFR2 signalling related to angiogenesis, provides new insights in NADPH oxidase and/or FPR1 as possible targets for therapies against angiogenesis-dependent diseases. The discovery of crosstalk between FPR1 and Flk-1/KDR provides further opportunities for drug discovery strategies for angiogenesis driven by an increase of VEGFR2 activity.

S. Franceschelli et al. examined the effects of phenethyl isothiocyanate (PEITC), one of the best-studied members of the organic isothiocyanates (ITC) family in the regulation of the cytoskeleton and morphology of endothelial cells (ECs). They demonstrated that PEITC induces PI3K/Akt promoting significant morphological changes in ECs that come back to the normal size and morphology after 16 hours from treatment. This effect was related to the activation of an important small GTPase protein Rac1. The activation of this latter led to JNK activation that promoted cytoskeleton remodelling influencing endothelial cell survival and growth. The authors showed that JNK promoted BAG3 overexpression. The expression of BAG3 corresponds to a recovery of cellular morphology after an initial damage by PEITC treatment; in fact, downregulation of Bag3 protein resulted in the loss of morphology recovery. In conclusion, they identified an important mechanism that led to actin refolding and the recovery of cell morphology which is driven by BAG3 that could represent an important target to recovery endothelial cells damage during cardiovascular diseases.

A. Fedorowicz et al. showed that in diabetic condition, increased ROS production may promote an increased permeability of pulmonary microcirculation related to NO impairment and increase compensatory levels of PGI2 and CD141 while the endothelial alteration detected in the aorta was associated with NO, PGI2, and CD141 impairment. This study highlighted various changes in pulmonary and peripheral endothelial function leading to endothelial dysfunction during diabetes.

The endothelial dysfunction is also a characteristic of patients affected by inflammatory bowel disease (IBD) such as Crohn's disease. In fact, IBD even in the absence of classic cardiovascular risk factors have a higher risk for endothelial dysfunction and atherosclerosis. In this context, A. G. Gravina et al. analysed the relationship between inflammation, oxidative stress, and endothelial dysfunction in IBD in order to predict the possible role of such inflammation in cardiovascular disease development. The authors focused their attention on pharmacological and nonpharmacological therapeutic targets, both of them aiming at attenuating chronic inflammation and oxidative stress.

Y. Shen and colleagues demonstrated that transplantation of bone marrow mesenchymal stem cells can be used to treat radiation-induced artery injury once again by suppressing oxidative stress and inflammation. Actually, the authors showed that this approach was able to upregulate antioxidant enzymes including HO-1 and catalase and downregulate inflammatory factors such as TNF-*α*, ICAM-1, and TGF-*β*.

C. R. Balistreri and colleagues found in patients with bicuspid valve disease a significant reduction of T and B lymphocyte cell subsets, compared to tricuspid aortic valve patients. This reduction, like those observed in old people, makes the individuals more vulnerable to chronic stress, inflammation, and oxidative stress, leading to cardiovascular diseases.

Interestingly, in a review article, G. Amodio et al. extensively highlighted the involvement of the unfolded protein response (UPR) pathways in the oxidative stress-induced endothelial dysfunction. In particular, authors underlined the importance of the endoplasmic reticulum (ER) source of ROS in the pathogenesis of cardiovascular diseases and the dual role of UPR, both prooxidant and antioxidant. They also reported the recent literature suggesting how the modulation of ER stress and UPR signalling may be a new therapeutic strategy to counteract the endothelial dysfunction. However, since some aspects on this field remain unclear, more investigations on animal models are encouraged. Identification of new molecular pathways aimed to develop therapeutic procedure based on pharmacological and nonpharmacological approaches, at different cellular levels, could represent an important milestone to reduce the onset of cerebral and cardiovascular diseases.

*Valeria Conti* *Maurizio Forte* *Albino Carrizzo*

